# Arginine Increases Tolerance to Nitrogen Deficiency in *Malus hupehensis* via Alterations in Photosynthetic Capacity and Amino Acids Metabolism

**DOI:** 10.3389/fpls.2021.772086

**Published:** 2022-01-14

**Authors:** Qi Chen, Yanpeng Wang, Zhijun Zhang, Xiaomin Liu, Chao Li, Fengwang Ma

**Affiliations:** State Key Laboratory of Crop Stress Biology for Arid Areas, Shaanxi Key Laboratory of Apple, College of Horticulture, Northwest A&F University, Xianyang, China

**Keywords:** arginine, *Malus*, nitrogen, metabolite, amino acid

## Abstract

Arginine plays an important role in the nitrogen (N) cycle because it has the highest ratio of N to carbon among amino acids. In recent years, there has been increased research interest in improving the N use of plants, reducing the use of N fertilizer, and enhancing the tolerance of plants to N deficiency. Here, the function of arginine in the growth of apple (*Malus hupehensis*) under N deficiency was explored. The application of 100 μmol L^–1^ arginine was effective for alleviating N-deficiency stress. Exogenous arginine promoted the absorption and use of N, phosphorus (P), and potassium (K) under low N stress. The net photosynthetic rate, maximal photochemical efficiency of photosystem II, and chlorophyll content were higher in treated plants than in control plants. Exogenous arginine affected the content of many metabolites, and the content of many amino acids with important functions was significantly increased, such as glutamate and ornithine, which play an important role in the urea cycle. Half of the metabolites were annotated to specialized metabolic pathways, including the synthesis of phenolic substances, flavonoids, and other substances with antioxidant activity. Our results indicate that arginine promotes the plant photosynthetic capacity and alters amino acid metabolism and some antioxidants including phenolic substances and flavonoids to improve the tolerance of apple to N deficiency, possibly through the improvement of arginine content, and the absorption of mineral.

## Introduction

Nitrogen (N) is an essential element for plant growth. In addition to being absorbed and utilized by plants, a large amount of N in soil is lost in the form of NO_2_, NO_x_, N_2_O, N_2_, dissolved organic N (DON), and particulate organic N (PON) (Zhang et al., 2015), which can lead to a gradual reduction of available N in soil. The use of N fertilizer can increase the proportion of N in soil and crop yields. Although the large amount of N fertilizer used in the agricultural industry increases the economic output of farmers, it can also lead to various problems such as water eutrophication and soil acidification (Guo et al., 2010; Liu et al., 2021). Therefore, improving the tolerance of plants to N-deficient conditions is an effective approach for mitigating soil N loss and environmental contamination caused by the excessive application of N fertilizer.

Nitrogen is involved in nearly all metabolic processes in plants. Although some plants use N-fixing microorganisms to obtain N, most plants meet their own growth and development needs by absorbing N from the soil. Therefore, low N availability in the soil can affect the metabolism, resource allocation, growth, and development of plants (Xu et al., 2012; Hsieh et al., 2018). The level of N absorbed by the plant directly affects the activity of enzymes involved in N absorption and transport in plants, and the activity of these enzymes is often closely related to chlorophyll (chl) synthesis. Therefore, chlorophyll is degraded when plants are faced with N deficiency, and plants often exhibit symptoms of leaf chlorosis and experience reductions in photosynthetic rate (Wang et al., 2019; Wen et al., 2019). Some plants also exhibit a decrease in height, the number of nodes, and leaf area (Chen et al., 2018). Previous studies have shown that N affects the activity of enzymes involved in the Calvin cycle, and a limited N supply reduces the CO_2_ assimilation capacity and decreases the light quantum yield (Φ) (Sun et al., 2016). A low N supply also affects the electron transport efficiency and maximum photochemical efficiency of PSII (*F_v_/F_m_*) (Luisa et al., 2018). Liu et al. (2020) found that the *F_v_/F_m_* of *Malus hupehensis* decreases markedly under N deficiency. In maize, Lu and Zhang (2000) also found that N deficiency affects plant PSII.

There are two forms of soil N uptake by plants: inorganic N (nitrates and ammonium salts) and organic N, mainly in the form of amino acids (Torgny et al., 2009). NO_3_^–^ uptake in plants is primarily achieved by NO_3_^–^ transporters, a family of transporters that can be divided into the low-affinity system and the high-affinity system based on their transport activity. The low-affinity transport system is mainly mediated by the NRT1 (nitrate transporter 1) protein, whereas the high-affinity transport system is mainly mediated by the NRT2 protein (Fan et al., 2017; Asif et al., 2019). Ammonium transport is also carried out by ammonium transporters (AMTs) (Bai et al., 2010). The transport of nitrates and ammonium salts has been extensively studied, but plants can also absorb and use organic N directly. Amino acid transporters are currently classified into three major groups: ATFs (amino acid transporter superfamily), APCs (amino acid-polyamine-choline transporter superfamily), and UMAMITs (usually multiple acids move in and out transporter superfamily). They are responsible for the absorption and transport of different types of amino acids (Pratelli and Pilot, 2014; Dinkeloo et al., 2018). For example, AtAAP5 (amino acid permease 5) plays an important role in the uptake of arginine and lysine at common concentrations in soil (Svennerstam et al., 2011). In addition, amino acid transporters exhibit some common characteristics, such as broad expression patterns and substrate specificity (Yang et al., 2020).

Arginine has the highest ratio of N to carbon among the 21 amino acids. The anabolism of arginine involves many metabolites and processes. Glutamic acid is the initial substrate for arginine synthesis (Winter et al., 2015); it thus provides a means of N storage and transport and has important functions in plants. Previous studies have shown that arginine metabolism is closely related to N metabolism, and many of the pathways in arginine metabolism show tissue specificity in plants (Gao et al., 2009). Ornithine is both a product and metabolic intermediary of arginine metabolite (Urbano-Gámez et al., 2020). These amino acids also play an important role in N utilization and the urea cycle in plants. Arginine succinate synthase (ASS), a key enzyme in arginine synthesis, has been reported to cause ASS1-deficient cell death in animals (Locke et al., 2016). Arginine can also undergo decarboxylation by arginine decarboxylase (ADC) and be converted to guanidine butylamine and then putrescine and other polyamines (Patel et al., 2017). Nitric oxide synthase catalyzes the conversion of arginine to citrulline and NO, and arginase (ARG) catalyzes the decomposition of arginine to ornithine and urea; urea is the substrate that links arginine metabolism and the urea cycle (Cao et al., 2010). The activity of these enzymes controls the direction of arginine metabolism.

Recent studies have focused on the function of arginine and related genes. Exogenous arginine treatment delays fruit coloring, inhibits fruit ripening, and increases the activity of antioxidant enzymes in strawberry fruits (Lv et al., 2020). Arginine also increases the N content in tomato (Wang et al., 2021) and NO content in wheat seedlings (Karpets et al., 2018). Overexpression of *OsARG* in rice (cv. Kitaake) increases the number of grains per plant under N deficiency (Ma et al., 2013). Infection of mature *Arabidopsis thaliana* with *Botrytis cinerea* up-regulates *ARGAH2* expression (Brauc et al., 2011). T-DNA insertional mutants of *argah1-1* and *argah2-1* and double mutants of *argah1argah2* show increased tolerance to abiotic stress, including water stress, salt stress, and low temperature, which is accompanied by the accumulation of NO and polyamines. Overexpression of arginase reduces tolerance to abiotic stress (Teresita et al., 2008; Shi et al., 2013). The *adc*-silenced line is more susceptible than parental WT plants to infection by *Botrytis cinerea* (Chávez-Martínez et al., 2020). Salt stress up-regulates the expression of *GHASS1* in cotton (Wang et al., 2016), and its expression peaks after 1 day of salt stress, suggesting that *GHASS1* might be involved in the early response of cotton to salt stress.

Research on arginine in *Malus* is still in an incipient stage. Arginine is an N storage substance that can alleviate N-deficiency stress, delay senescence, and reduce the use of N fertilizer. The aim of this study was to characterize changes in the physiological parameters and exogenous substances of *Malus hupehensis* Rehd. under N deficiency and explore the physiological functions and mechanisms of arginine in plants under low N stress. Overall, the results of our study have implications for the apple industry, especially the development of the green economy, environmental protection, and sustainability.

## Materials and Methods

### Materials and Growth Conditions

The experiment was carried out at Northwest A&F University, Yangling (34° 15′ N, 108° 4′ E) Shaanxi, China. Seeds of *M. hupehensis* were collected in Pingyi (35° 70′ N, 117° 25′ E), Shandong Province. The seeds were disinfected and stored in sand for 2 months at 4°C to break dormancy. The germinated seeds were seeded in a nursery (12 cm × 12 cm). Plants of similar size were used for hydroponics and grown in 8–10 nurseries. Hoagland nutrient solution was the source of nutrients during hydroponics. The two N concentrations applied were 5 mmol L**^–^**^1^ and 0.2 mmol L**^–^**^1^. N was provided exclusively in the form of NO_3_**^–^**. Arginine was added 3 days before N-deficiency treatment to facilitate the adaptation of plants to the experimental environment. The temperature was maintained at 23–25°C in the daytime and 15–18°C at night. The sodium lamp provided light during the 14/10 h light/dark photoperiod (photon flux density was 160 mmol m**^–^**^2^ s**^–^**^1^). Oxygen was added to the nutrient solution to maintain the oxygen concentration at 8.0–8.5 mg L**^–^**^1^ through the dissolved oxygen controller (FC-680; Corporation of Super, Shanghai, China). The nutrient solution was renewed every 5 days.

### Hydroponics Screening

The plants were pre-cultivated for 15 days to permit adaptation to hydroponics conditions. To determine the optimal concentration of exogenous arginine, the plants were divided into seven treatments at 20 days: half-strength nutrient solution (5 mmol L**^–^**^1^) and N-deficiency treatment with 20, 100, 500, 2500, and 10000 μmol L**^–^**^1^ arginine. Arginine was supplemented throughout the entire experiment. The net photosynthetic rate (*Pn*) was measured at Day 0, 5, 10, 15, 20, and 25 after treatment; the dry weight, fresh weight, and plant height of all groups were measured at 25 days after treatment; and the phenotypic characteristics of the plants were recorded.

According to the optimal arginine concentration, the plants were divided into four treatments: control (1/2 Hoagland solution containing 5 mmol L**^–^**^1^ N, CK), control + arginine (1/2 Hoagland solution containing 5 mmol L**^–^**^1^ N with 100 μmol L**^–^**^1^ arginine, CKA), N-deficiency solution (1/2 Hoagland solution containing 0.2 mmol L**^–^**^1^ N, LN), and LN + arginine (1/2 Hoagland solution containing 0.2 mmol L**^–^**^1^ N with 100 μmol L**^–^**^1^ arginine, LNA). The physiological indexes of each treatment were measured.

### Growth Measurements

The shoot height (SH) was measured from the top of the stem to the stem base; the leaf number was counted per plant at 20 days after treatment. On the first and last day of the treatments, the plants were divided into leaves, stems, and roots for weighing. The samples were then washed with tap water, distilled water, and double water, followed by fixation at 105°C for 30 min and drying at 72°C until a constant weight was achieved (at least 72 h). The fresh weight and dry weight were calculated from the weight of the leaves, stems, and roots before and after drying, respectively.

### Measurement of Malondialdehyde Content and Root Activity

The malondialdehyde (MDA) content was measured per the instructions provided in an MDA reagent kit (Suzhou Comin Biotechnology Co., Ltd., China). Root activity was measured using triphenyl tetrazolium chloride (TTC) following the method of Joseph et al. (2010).

### Determination of Nitrogen, Potassium, and Phosphorus Concentrations

The tissues in the four treatments were collected at 20 days after treatment to estimate the N, P, and K concentrations. After quick-freezing, tissue (0.1 g) was ground and digested with concentrated sulfuric acid (H_2_SO_4_, AR, 98%) and hydrogen peroxide (H_2_O_2_, GR, ≥30%). The N and P content was measured by an AutoAnalyzer 3 continuous-flow analyzer (AA3; SEAL Analytical, Norderstedt, Germany), and the K content was measured by a flame photometer (M410; Sherwood Scientific, Cambridge, United Kingdom) according to the method of Li et al. (2013).

### Determination of Nutrient Uptake Fluxes, Transport, Accumulation, and Partitioning

The formulas for nutrient uptake fluxes (TN) are as follows (Kruse et al., 2007):


$${\text{TN}}_{\text{r}}=\,\text{RGR}\times {\text{DW}}_{\text{r}}\times {\text{C}}_{\text{r}}$$

$${\text{TN}}_{\text{s}}=\,\text{RGR}\times {\text{DW}}_{\text{s}}\times {\text{C}}_{\text{s}}$$

$${\text{TN}}_{\text{l}}=\,\text{RGR}\times {\text{DW}}_{\text{l}}\times {\text{C}}_{\text{l}}$$

where, RGR is the relative growth rate, DW is the dry weight, and C is the concentration of elements in each tissue. The uptake flux was expressed in units of milligrams per plant per day or micrograms per plant per day.

The transportation of N, P, and K was defined as the total amount of element transported to leaf and stem tissue per gram root DW per day. The accumulation of N, P, and K in the roots was defined as the total amount of nutrient absorbed into root per gram root DW per day. The formula is as follows (Kruse et al., 2002):


$$\text{transfer}/\text{accumulation}=\left({\text{M}}_{2}-{\text{M}}_{1}\right)\times \left({\text{LnW}}_{2}-{\text{LnW}}_{1}\right)/\left({\text{W}}_{2}-{\text{W}}_{1}\right)/\left({\text{T}}_{2}-{\text{T}}_{1}\right)$$

where, M represents the total content of elements, W represents the dry weight of roots, and T is the treatment time.

The element content of each part (root, stem, and leaf) is the product of the dry weight of each part and the element concentration.

### Enzyme Activity Assays

The tissues of four treatments were collected at 20 days after treatment. The samples were ground into powder with liquid N and stored at –80°C. Samples were weighed (0.1 g) for each enzyme activity measurement. The activity of nitrate reductase (NR), nitrite reductase (NiR), glutamine synthetase (GS), and glutamate synthase (GOGAT) was measured per the instructions provided by corresponding kits (Suzhou Comin Biotechnology Co., Ltd., China). Absorbance of NR and GOGAT was recorded at 340 nm. Absorbance of NIR and GS was recorded at 540 nm. The standard was based on the instructions provided by the kits. NR activity was expressed as catalytic reduction of 1 nmol NADH per min per g of fresh weight. NiR activity was expressed as the reduction of 1 μmol NO_2_**^–^** per g tissue per hour. GS activity was expressed as 1 μmol of γ-glutamyl hydroxamic acid produced per gram of tissue per ml of reaction system per hour. GOGAT activity was expressed as 1 nmol of NADH consumed per gram of tissue per minute.

### Measurement of Photosynthetic Characteristics

Chl was extracted with 80% acetone at 0 and 20 days. The total chl, chl *a*, chl *b*, and carotenoid (car) content was measured spectrophotometrically following the procedure of Arnon (1949) with minor modifications.

The *Pn*, stomatal conductance (*gs*), intercellular carbon dioxide concentration (*Ci*), and transpiration rate (*Tr*) were measured with a portable photosynthesis system (CIRAS3) from 9:00 am to 10:00 am. All measurements were performed at 1000 μmol photons m^–2^ s^–1^ and a constant airflow rate of 500 μmol s^–1^. The cuvette CO_2_ concentration was set to 400 μmol CO_2_ mol^–1^ air, with a vapor pressure deficit of 2.0–3.4 kPa. Measurements were taken from the fully extended leaves located at the same position from five randomly selected plants in each group.

Chl fluorescence was measured by a Dual-PAM-100 Chlorophyll Fluorometer (Walz, Germany). After 30 min of dark adaptation, the minimum fluorescence (*F*_0_) and maximum Chl fluorescence yield (*F_m_*) were measured. *F_v_/F_m_* was calculated as (*F_m_−F_0_*)/*F_m_*.

### RT-PCR Analysis

Total RNA was extracted from leaves using a Wolact^®^ Plant RNA Isolation Kit (Vicband, HongKong, China) per the manufacturer’s instructions. qRT-PCR was performed on a LightCycler 480 (Roche, Indianapolis, IN, United States) Real-Time System using SYBR Premix Ex Taq II (Takara, Kyoto, Japan) following a previously described method (Gao et al., 2020). Sequences of the primers for *MdAMT1;2, MdAMT2;1*, M*dFd-GOGAT*, and *MdNADH-GOGAT* were designed with Primer Premier 5 software (Biosoft International, Palo Alto, CA, United States) (Supplementary Table 1). *MdMDH* was used as an internal reference gene.

### Metabolomic Analysis

Biological samples were crushed using a mixer mill (MM 400, Retsch). Extraction and detection methods were adjusted following the method of Chen et al. (2013). Briefly, 100 mg of powder was dissolved with 1.2 mL of 70% methanol solution. The solution was vortexed for 30 s every 30 min a total of 6 times. The sample was extracted at 4°C overnight, followed by centrifugation at 12,000 rpm for 10 min. The supernatant was filtrated through a 0.22-μm organic strainer before UPLC-MS/MS (UPLC, SHIMADZU Nexera X2; MS, Applied Biosystems 4500 Q TRAP) analysis. The UPLC was equipped with an Agilent SB-C18 column (1.8 μm, 2.1 mm × 100 mm). The mobile phase consisted of solvent A, pure water with 0.1% formic acid, and solvent B, acetonitrile with 0.1% formic acid. The gradient program was as follows: 0–9 min, 95% A to 5% A and 5% B to 95% B with a linear gradient and hold for 1 min; 5% A to 95% A and 95% B to 5% B in 1.10 min and hold for 2.9 min. The flow velocity was 0.35 mL min**^–^**^1^, the column temperature was 40°C, and the injection volume was 4 μL. The effluent was alternatively connected to an ESI-triple quadrupole-linear ion trap (QTRAP)-MS.

LIT and triple quadrupole (QQQ) scans were acquired on a triple quadrupole-linear ion trap mass spectrometer (Q TRAP), AB4500 Q TRAP UPLC/MS/MS System, equipped with an ESI Turbo Ion-Spray interface, operating in positive and negative ion mode and controlled by Analyst 1.6.3 software (AB Sciex). The ESI source operation parameters were as follows: ion source, turbo spray; source temperature, 550°C; ion spray voltage (IS), 5500 V (positive ion mode)/-4500 V (negative ion mode); ion source gas I (GSI), gas II (GSII), and curtain gas (CUR) were set at 50, 60, and 25.0 psi, respectively; and the collision-activated dissociation (CAD) was high. Instrument tuning and mass calibration were performed with 10 and 100 μmol L**^–^**^1^ polypropylene glycol solutions in QQQ and LIT modes, respectively. QQQ scans were acquired as MRM experiments with collision gas (N) set to medium. DP and CE for individual MRM transitions were done with further DP and CE optimization. A specific set of MRM transitions were monitored for each period according to the metabolites eluted within this period.

### Determination of Amino Acids

The amino acids were extracted and detected by LC-MS following the method of Jin et al. (2019). Fresh tissue was ground in liquid N, 500 mg of sample powder was weighed in tubes, and 1 mL of 50% ethanol (containing 0.1 mol L**^–^**^1^ HCl) was added to tubes to extract amino acids. The supernatant was transferred to a new tube after centrifugation at 13,000 *g* and 4°C for 10 min. The liquid was filtered through a 0.22-μm organic strainer. The filtrate was diluted 20 times with methanol (AR, 98%) to a 1-mL volume. The LC-MS analysis was carried out on an Inertsil OSD-4C18 column (150 mm × 3.0 mm; packing size, 3.5 μm) at 25°C; 0.5% formic acid water (solvent A) and methanol (solvent B) were used as the mobile phase at a flow rate of 0.3 mL min**^–^**^1^. The content was calculated according to the standard curve.

### Statistical Analysis

Data on the content of metabolites detected were used for principal component analysis (PCA), hierarchical cluster analysis, and orthogonal partial least squares-discriminant analysis in R^1^ to analyze the accumulation of metabolites in response to arginine and N deficiency (Wang et al., 2015). The Pearson correlation coefficients were calculated in R to verify the repeatability between classes. Significantly regulated metabolites were filtered using the following criteria: VIP ≥ 1 and Log2FC (fold change) ≥ 2 or Log2FC ≤ 0.5. To avoid overfitting, a permutation test (200 permutations) was performed. To clarify the role of arginine in response to N-deficiency stress, the identified metabolites were annotated using Kyoto Encyclopedia of Genes and Genomes (KEGG) pathway analysis. MSEA (metabolite sets enrichment analysis) was then conducted on the pathways with significantly regulated metabolites, and the significance of these pathways was determined by hypergeometric tests. Annotated metabolites were mapped to the KEGG Pathway database.

The data were statistically analyzed by one-way ANOVA. Tukey’s multiple range test was used to determine significant differences between means (*P* < 0.05) using SPSS 25.0 software (IBM Corp., Armonk, NY, United States).

## Results

### Hydroponics Screening

**FIGURE 1:**
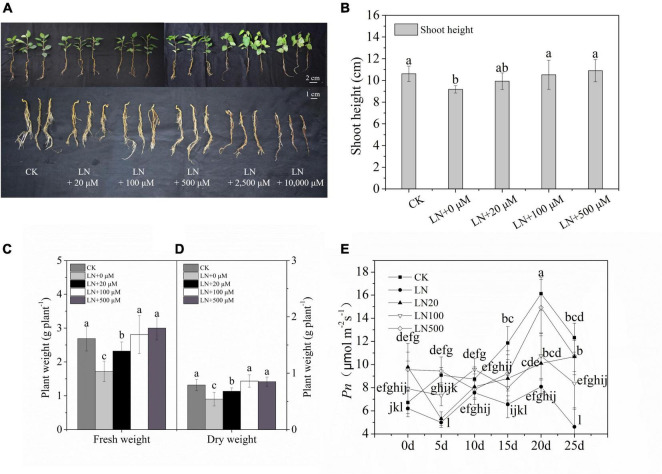
Effect of N deficiency on the growth parameters of *Malus hupehensis*. Whole plant and underground phenotypes **(A)**, shoot height (cm) **(B)**, fresh weight (g**^–^**^1^ plant) **(C)**, dry weight (g**^–^**^1^ plant) **(D)**, and net photosynthetic rate (*Pn*) **(E)** from 0 to 25 days every 5 days. CK, half-strength nutrient solution; LN, N-deficiency treatment; LN + 20 μmol L**^–^**^1^, N-deficiency treatment plus 20 μmol L**^–^**^1^ arginine; LN + 100 μmol L**^–^**^1^, N-deficiency treatment plus 100 μmol L**^–^**^1^ arginine; LN + 500 μmol L**^–^**^1^, N-deficiency treatment plus 500 μmol L**^–^**^1^ arginine; LN + 2500 μmol L**^–^**^1^, N-deficiency treatment plus 2500 μmol L**^–^**^1^ arginine; and LN + 10000 μmol L**^–^**^1^, and N-deficiency treatment plus 10000 μmol L**^–^**^1^ arginine. The picture was taken on the third d of the experiment). Values are means of ten replicates ± SD. Values not represented by the same letter are significantly different according to Tukey’s multiple-range test (*P* < 0.05).

The growth rate of apple was significantly inhibited under N-deficiency conditions. The growth of apple treated with 2500 and 10000 μmol L**^–^**^1^ arginine was severely inhibited, as indicated by the lower plant length, wilted leaves, absence of root growth, and even death on the 3rd d with 10,000 μmol L**^–^**^1^ arginine and 6th d with 2,500 μmol L**^–^**^1^ arginine (Figure 1A) under N-deficient solutions. Apple treated with 100 and 500 μmol L**^–^**^1^ arginine had better growth performance, indicating that this concentration of arginine alleviated the inhibition induced by N-deficiency stress (Figure 1).

Under N deficiency, the dry weight and *Pn* decreased sharply. However, the dry weight was 59.26% higher in apple plants treated with 100 μmol L**^–^**^1^ arginine than under N deficiency (Figure 1D). Exogenous application of 100 or 500 μmol L**^–^**^1^ arginine resulted in higher *Pn* values during treatments compared with the control (Figure 1E). Based on these results, 100 μmol L**^–^**^1^ arginine was the optimal concentration used in subsequent experiments.

### Exogenous Arginine Enhanced the Growth of *Malus hupehensis* Under Nitrogen Deficiency

**FIGURE 2:**
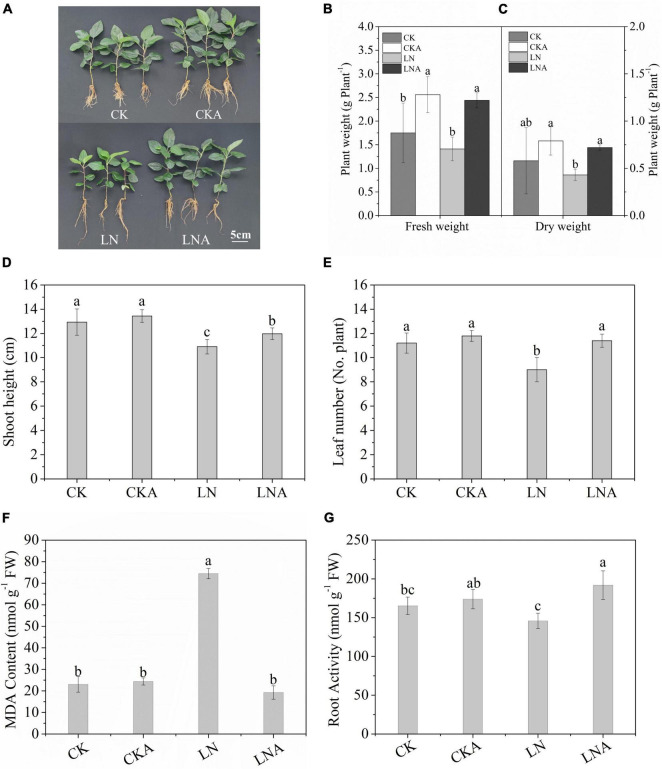
Analysis of the growth parameters of CK, CKA, LN, and LNA at 20 days. We measured the phenotype **(A)**, fresh weight (g^–1^ plant) **(B)**, dry weight (g^–1^ plant) **(C)**, shoot height (cm) (No. plant) **(D)**, and the number of leaves **(E)** of apple plants from these four treatments. The content of MDA after 20 days **(F)**. Root activity of CK, CKA, LN, and LNA after 20 days of treatment **(G)**. Half-strength nutrient solution (CK); half-strength nutrient solution + 100 μmol L^–1^arginine (CKA); N-deficiency solution (LN); and N-deficiency solution + 100 μmol L^–1^ arginine (LNA). Values are means of five replicates ± SD. Values not represented by the same letter are significantly different according to Tukey’s multiple-range test (*P* < 0.05).

The growth of *M. hupehensis* plants was inhibited significantly under N deficiency, and growth was restored via exogenous arginine application (Figure 2A). The dry weight and fresh weight of *M. hupehensis* plants were higher under exogenous arginine application compared with LN (Figures 2B,C); the growth of plants was also enhanced by arginine application under a normal nutrient supply. The SH and number of leaves were lower under N deficiency compared with when a normal level of N was supplied (Figures 2D,E). There were no significant differences observed between CK and CKA.

Root vitality of *M. hupehensis* was significantly affected by N deficiency. Root vitality was higher for plants under LNA compared with plants under LN (Figure 2G).

The MDA content was higher in LN than in LNA, and no clear differences were observed among CK, CKA, and LNA (Figure 2F). This indicated that arginine alleviates oxidative damage to the cell membrane.

These results indicated that exogenous arginine enhanced aboveground growth and root activity under N deficiency and alleviated the damage to cells caused by N-deficiency.

### Concentration, Transport, Accumulation, and Partitioning of Nitrogen, Potassium, and Phosphorus

Nutrient concentrations in different tissues were altered at 20 days after N-deficiency treatment. Arginine increased the total N concentration of the leaves, stems, and roots under stress (Supplementary Figures 1A–C). In the leaves and roots (Supplementary Figures 1D,F), the total K concentration was higher under LNA than under LN, but in the stems (Supplementary Figure 1E), the total K concentration was higher under LN compared with CK. The total P concentration in the stems and roots increased after the application of arginine under N stress (Supplementary Figures 1H,I). The leaf P content was lower under LNA than under LN (Supplementary Figure 1G).

**FIGURE 3:**
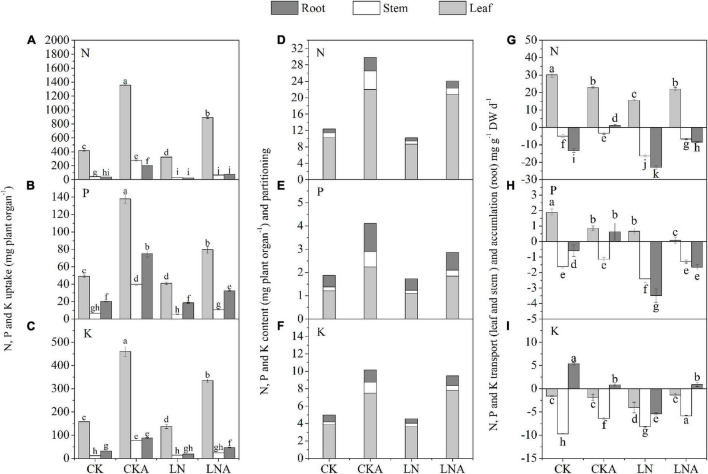
Effects of arginine on N, P, and K in *M. hupehensis* under low N. The uptake of total N **(A)**, K **(B)**, and P **(C)** in the leaves, stems, and roots. The accumulation and partitioning of N **(D)**, P **(E)**, and K **(F)** and transport of N **(G)**, P **(H)**, and K **(I)** in the leaves, stems, and roots. Values are means of three replicates ± SD. Values not represented by the same letter are significantly different according to Tukey’s multiple-range test (*P* < 0.05).

The uptake, accumulation, and partitioning of N, P, and K were affected under N deficiency. The uptake of N, P, and K was decreased under N deficiency in *M. hupehensis*. The use of arginine significantly alleviated this inhibition (Figures 3A–C).

The content of N, P, and K was highest in leaves and lowest in stems. The application of arginine significantly increased the content of N, P, and K in the roots, stems, and leaves of *M. hupehensis*, especially the stems. Compared with LN, arginine also increased the N, P, and K content in tissues of *M. hupehensis* under low N stress (Figures 3D–F).

Under N deficiency, arginine increased the accumulation of N, P, and K in the roots, as well as the rate of transport of these nutrients in the stems compared with LN. The N and P transport rate to leaves was decreased, and the N and K transport rate was increased (Figures 3G–I). These results indicated that exogenous arginine treatment had a significant effect on nutrient transport, accumulation, and partitioning under N-deficiency stress.

### Changes in Nitrogen Metabolism-Related Enzyme Activity and Gene Expression

**FIGURE 4:**
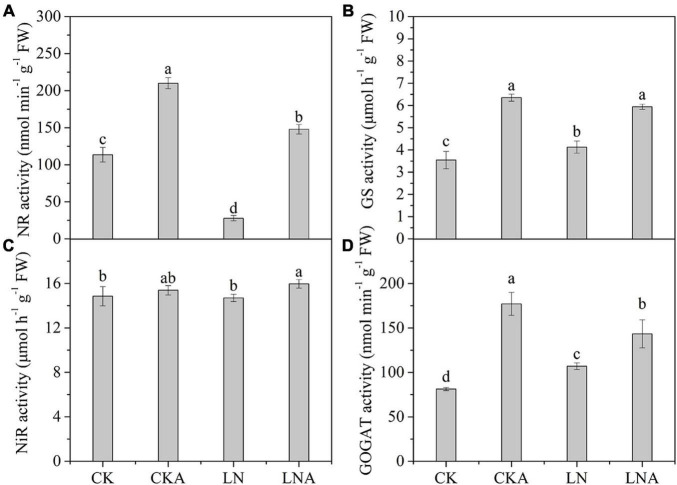
The activity of enzymes in the leaves of CK, CKA, LN, and LNA after 20 days. NR activity **(A)**, GS activity **(B)**, NiR activity **(C)**, and GOGAT activity **(D)**. Values are means of five replicates ± SD. Values not represented by the same letter are significantly different according to Tukey’s multiple-range test (*P* < 0.05).

**FIGURE 5:**
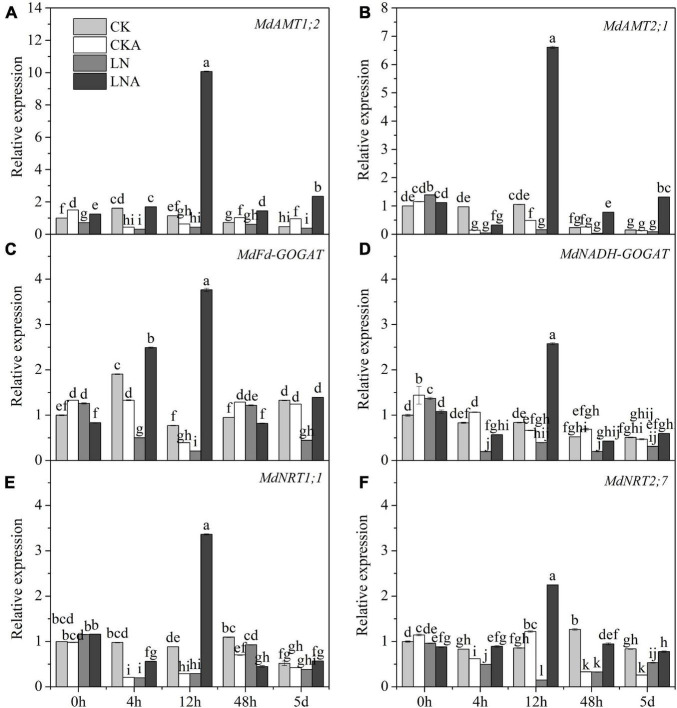
The expression level of N metabolism enzymes. *MdAMT1;2* **(A)**, *MdAMT 2;1* **(B)**, *MdFd-GOGAT* **(C)**, and *MdNADH-GOGAT* **(D)**, *MdNRT1;1* **(E)** and *MdNRT2;7* **(F)** in CK, CKA, LN, and LNA after treatment. The data are shown up to the fifth day, as no significant responses of the genes were observed after 5 days. Values are means of three replicates ± SD. Values not represented by the same letter are significantly different according to Tukey’s multiple-range test (*P* < 0.05).

Given the observed effects of arginine on the absorption and distribution of nutrients, we measured the activity of these four enzymes. The activity of these four enzymes was significantly inhibited under N-deficiency stress, and exogenous arginine alleviated this inhibition (Figure 4). The ammonium transporter genes *MdAMT1;2* and *MdAMT2;1* and the ammonium assimilation genes *MdFd-GOGAT* and *MdNADH-GOGAT* were significantly up-regulated at 12 h after low N stress with arginine application (Figure 5); the NO_3_**^–^** transporter genes *MdNRT1;1* and *MdNRT2;7* were up-regulated 3.3 and 2.3 times, respectively, at 12 h, and no significant response was observed after 12 h.

### Changes in Photosynthetic Parameters and Chlorophyll

**FIGURE 6:**
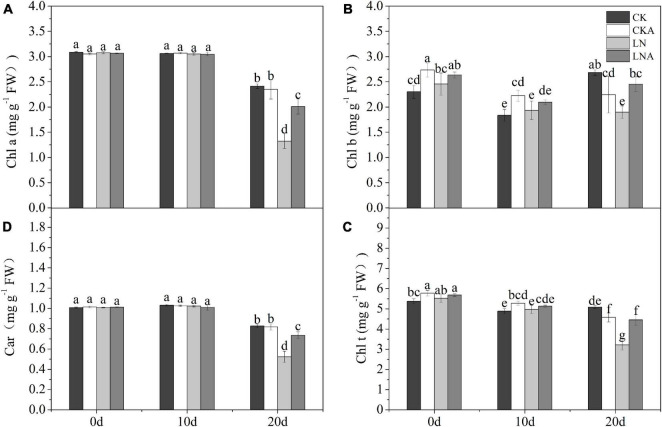
Analysis of chlorophyll. Chlorophyll *a* **(A)**, chlorophyll *b* **(B)**, total chlorophyll **(C)**, and carotenoids **(D)**. Values are means of five replicates ± SD. Values not represented by the same letter are significantly different according to Tukey’s multiple-range test (*P* < 0.05).

The chl *a*, chl *b*, car, and total chl content under N deficiency decreased significantly at 20 days after treatment; chl *b* was most affected by N deficiency (Figure 6B). The chl *a*, chl *b*, car, and total chl content was higher under LNA than under LN (Figure 6).

**FIGURE 7:**
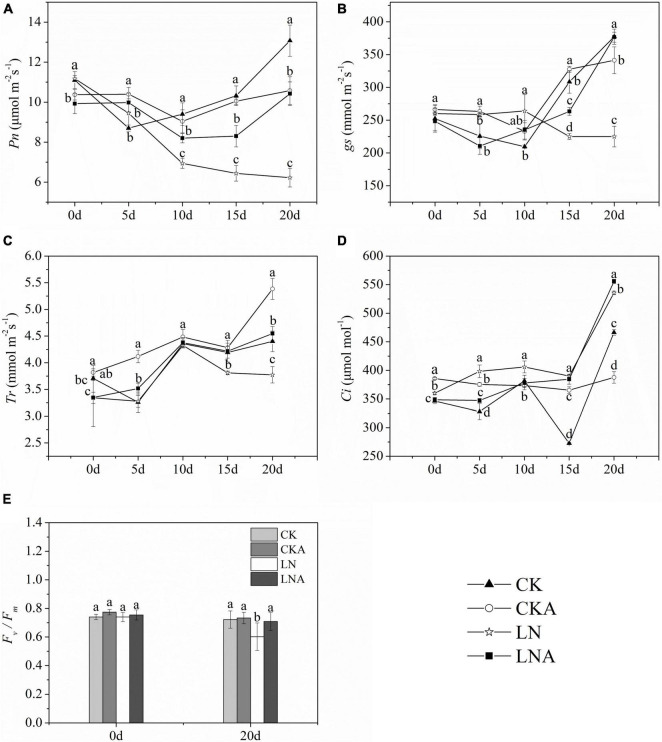
Changes in the photosynthetic system of *M. hupehensis.* Net photosynthetic rate (*Pn*, **A**), stomatal conductance (*gs*, **B**), transpiration rate (*Tr*, **C**), intercellular CO_2_ concentration (*Ci*, **D**), and *F_v_/F_m_* **(E)** of CK, CKA, LN, and LNA after 20 days. Values are the means of ten replicates ± SD. Values not represented by the same letter are significantly different according to Tukey’s multiple-range test (*P* < 0.05).

The *Pn*, *gs*, and *Tr* of plants decreased at 20 days after low N treatment (Figures 7A–C). However, *Pn* was higher under LNA than under LN. Under normal nutrient conditions, *Pn* was higher under CK than under CKA at 20 days (Figure 7A). Starting on the fifth day, the *Tr* of LNA was higher than that of LN, and *gs* was higher under LNA than under LN on the 10 days (Figures 7B,C). Lower *Ci* aided the ability of plants to cope with stress, and *Ci* was high under LN (Figure 7D), indicating that plants were under greater nutrient stress; *Ci* was low under CK, CKA, and LNA.

*F_v_/F_m_* measurements indicated that *F_v_/F_m_* was significantly lower under LN than under CK and LNA, but no significant differences between CKA and CK were observed (Figure 7E). The application of exogenous arginine alleviated damage to the photosynthetic system under low N stress but had no effect under normal N conditions.

### Differential Metabolite Profiling Analysis

**FIGURE 8:**
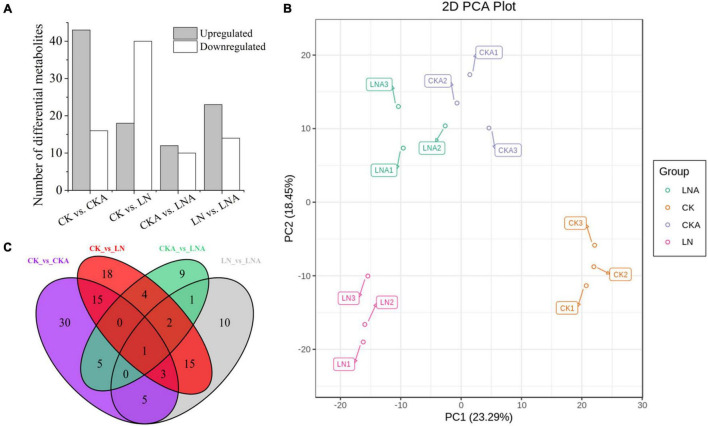
Differential metabolite analysis. Number of differential metabolites for the comparisons CK vs. CKA, CK vs. LN, CKA vs. LNA, and LN vs. LNA **(A)**. PCA score diagram of each group. PC1 represents the first principal component, and PC2 represents the second principal component **(B)**. Venn diagram of the common metabolites and differential metabolites among the four groups **(C)**. Significantly regulated metabolites were those with fold change ≥ 2 or fold change ≤ 0.5 and VIP ≥ 1.

We analyzed the differential metabolites of *M. hupehensis* plants. PCA was used to conduct an unsupervised clustering analysis of the detected metabolites. The replications of the different treatments were clustered, and there was clear separation among the four treatments, indicating that the results were reliable. LN and CK were significantly separated along the first principal component (PC1), accounting for 23.29% of the total variance. LN and LNA were significantly separated along the second principal component (PC2), accounting for 18.45% of the total variance (Figure 8B). Differences between CKA and LNA along PC1 and PC2 were small (Figure 8B).

A total of 59, 58, 22, and 37 differential metabolites were identified in the four comparison groups (CK vs. CKA, CK vs. LN, CKA vs. LNA, and LN vs. LNA, respectively) (Figure 8A). In the CK vs. LN and LN vs. LNA comparisons, 18 and 23 metabolites were up-regulated, and 40 and 14 metabolites were down-regulated, respectively (Supplementary Tables 2, 3). A total of 43 down-regulated and 16 up-regulated metabolites were identified between CK and CKA, and 10 down-regulated and 12 up-regulated metabolites were identified between CKA and LNA (Figure 8A). Venn diagram analysis showed that only one differential metabolite was present in all four comparison groups (Figure 8C).

**FIGURE 9:**
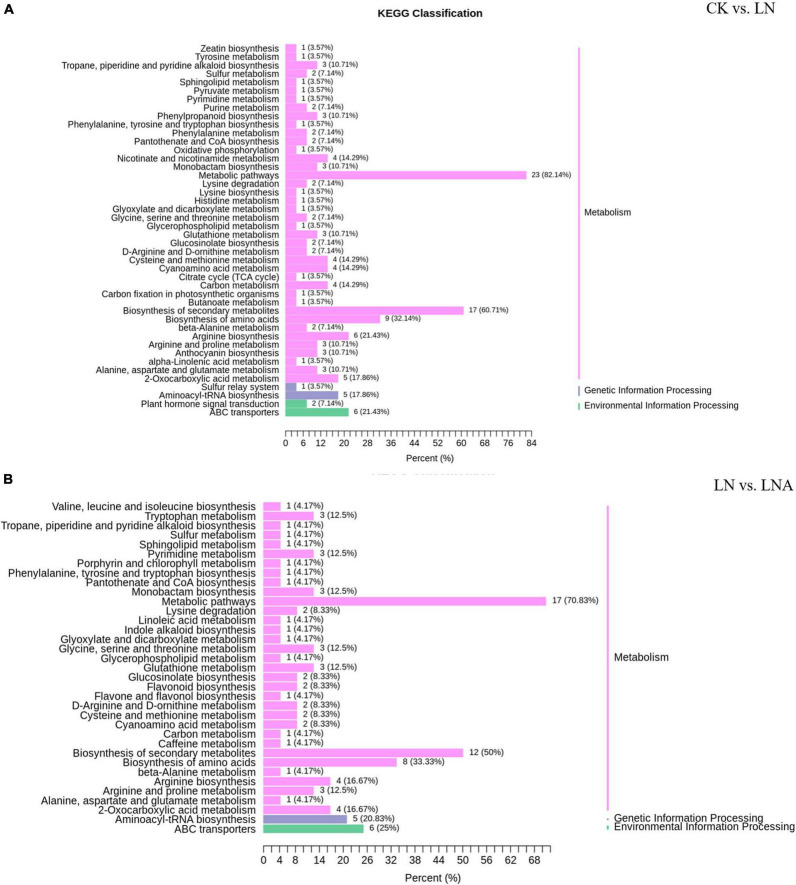
KEGG classification of differential metabolites. CK vs. LN **(A)** and LN vs. LNA **(B)**. The ordinate shows the KEGG metabolic pathways, and the abscissa shows the number of metabolites annotated to these pathways and the proportion of the number of metabolites to the total number of metabolites annotated.

The differential metabolites detected under N deficiency were annotated to several pathways through KEGG analysis. Most of the metabolites were annotated to the metabolism and biosynthesis of specialized metabolites, which accounted for 82.14% and 60.71% of total metabolites. Approximately, 1/3 of the metabolites were related to amino acid synthesis (Figure 9A). N deficiency also affected lysine synthesis and degradation, as well as histidine, arginine, serine, threonine, glycine, alanine, proline, and ornithine synthesis (Figure 9A). Some metabolites were involved in carbon and sulfur metabolism, the TCA (Tricarboxylic Acid Cycle) cycle, and carbon fixation in photosynthetic organisms, which are important for plant growth. Nicotinate and nicotinamide, oxidative, phosphorylation, anthocyanin, and 2-oxocarboxylic acid metabolism and synthesis were also related to metabolites under N deficiency.

Thirty-seven differential metabolites were detected in the LN and LNA comparison: a total of 23 up-regulated metabolites, including 14 amino acids and derivatives, 2 nucleotides and derivatives, 3 flavonoids, 2 alkaloids, 1 phenolic acid, and 1 organic acid, and a total of 14 down-regulated metabolites, including 2 amino acids, 3 flavonoids, 1 phenolic acid, 2 lipids, 3 terpenoids, and 3 alkaloids (Supplementary Tables 3, 4). Most were annotated to the metabolism and biosynthesis of specialized metabolites, which accounted for 70.83% and 50% of total metabolites, respectively. Metabolites related to indole alkaloid, linoleic acid, and caffeine were detected in LNA (Figure 9B).

Next, we compared the differential metabolites between CK vs. LN and LN vs. LNA. A total of 17 metabolites detected in both showed opposite expression patterns, including 12 amino acids (L-serine, 5-aminovaleric acid, pipecolic acid, L-asparagine, L-ornithine, L-citrulline, L-cysteinyl-L-glycine, L-arginine, N-monomethyl-L-arginine, homoarginine, glutathione-reduced form, and L-homomethionine), 3 flavonoids (delphinidin-3-O-arabinoside, malvidin-3-O-arabinoside, and kaempferol-3-O-rhamnosyl (1→2) glucoside), 1 organic acid (3-ureidopropionic acid), 1 phenolic acid (4-O-methylgallic acid), 1 alkaloid (diethanolamine), 2 terpenoids (1-oxo-siaresinolic acid, myrianthic acid), and 1 lipid (LysoPC 20:4). These metabolites were up-regulated in LNA and down-regulated in LN, with the exception of terpenoids (Supplementary Table 3). Other metabolites were only related to synthesis under N deficiency (Supplementary Table 2), suggesting that exogenous arginine did not affect these pathways.

### Exogenous Arginine Altered the Content of Endogenous Amino Acids

**FIGURE 10:**
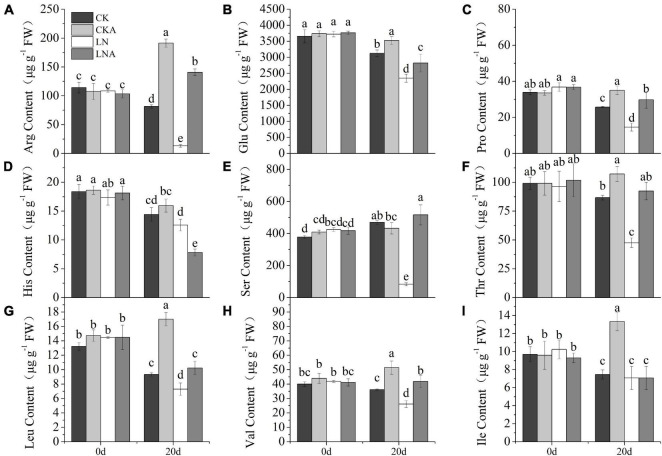
The content of amino acids. Arginine **(A)**, glutamic acid **(B)**, proline **(C)**, histidine **(D)**, serine **(E)**, threonine **(F)**, leucine **(G)**, valine **(H)**, and L-isoleucine **(I)** in CK, CKA, LN, and LNA on the 20th day of treatment. Values are means of five replicates ± SD. Values not represented by the same letter are significantly different according to Tukey’s multiple-range test (*P* < 0.05).

The content of arginine, glutamic acid, proline, serine, threonine, leucine, and valine was decreased (Figures 10A–C,E–H). However, the application of arginine restored the synthesis of these amino acids. The content of amino acids was higher in *M. hupehensis* growing under normal N conditions after arginine application. The content of histidine showed the opposite pattern: histidine content was high under LN but low when 100 μmol L**^–^**^1^ arginine was added at 20 days after N deficiency (Figure 10D). Under normal N conditions, the content of histidine was similar to that of other amino acids. The content of isoleucine significantly increased only in CKA (Figure 10I).

## Discussion

Nitrogen plays a prominent role in plant growth and development. Thus, the challenge of improving N use efficiency while reducing the use of N fertilizer has become a major focus of research. Arginine is known to be an important component of the N pool, and previous studies have shown that arginine is involved in the response to stress, including both biotic and abiotic stresses. The aim of this study was to investigate the role of arginine in mediating N-deficiency stress. Consistent with previous studies (Ji et al., 2018; Conceição et al., 2021), we found that the effect of arginine was dose-dependent. The photosynthetic rate of plants was higher under N deficiency when 100 μmol L**^–^**^1^ arginine was applied; these plants also exhibited high SH, dry weight, and numbers of leaves. These results indicate that arginine can alleviate N-deficiency stress.

Nitrogen, potassium, and phosphorus are three essential elements for plant growth. N has been shown to compose macromolecules such as nucleic acids, nucleoprotein, chl, and protein; thus, more N is required compared with other elements during plant growth and development (Wen et al., 2020). The absorption of N and P is coupled and affects plant growth; N and K are important for ensuring plant yields (Clemente et al., 2013; Yan et al., 2015). In our study, the level of P and K was affected by arginine. Increased K and P content was observed following arginine application. Under N-deficiency conditions, N absorption was inhibited, and N accumulated in the roots. However, the use of arginine under N deficiency enhanced the absorption and content of N, as well as the accumulation of N in the leaves and stems. Arginine is closely related to the storage and re-mobilization of N. Arginine dihydrolase AgrE/ArgZ of Anabaena, coupled with bifunctional proline oxidase PutA, mediates the conversion of arginine to glutamate, which provides a more direct route linking the arginine and ammonia pool (Zhang and Yang, 2019). After the genes controlling arginase synthesis were silenced in A*rabidopsis*, the nitric oxide synthase activity and increase in the NO content caused a decrease in N in plants (Flores et al., 2008). The total N concentration and content in the four treatments increased under arginine application. Arginine also facilitated nutrient transport and partitioning under N deficiency. NR and NiR are important enzymes in the process of nitrate reduction (Jung et al., 2021). GS and GOGAT are important enzymes involved in the process of ammonium assimilation (Esen and Öztürk Ürek, 2014). However, N transformation and key metabolic enzyme genes were up-regulated after arginine application under N deficiency at 12 h. Enzyme activity also increased at 20 days. There was a difference in the timing of the responses of genes and enzyme activity. Our results indicate that arginine can not only be transformed into N for plant absorption and use but can also promote the absorption and use of limited N, P, and K by plants.

Photosynthesis is sensitive to environmental changes; thus, the photosynthetic activity of plants can be altered by stress, including N deficiency. Most N is located in the chloroplasts of leaves and participates in the synthesis of photosynthetic-related substances. Approximately, 80% of the N in C3 plants is in the chloroplasts (Makino et al., 2003; Hiroyuki and Amane, 2018). In poplar, *Pn* is directly proportional to the N content (Luo and Zhou, 2019). In our experiment, the *Pn* of plants in low N environments was significantly decreased; *gs* and *Tr* also decreased under N deficiency. The application of exogenous arginine significantly alleviated the effect of N deficiency on plant photosynthesis and increased *Pn*. This effect may stem from the high N to carbon ratio in arginine. Arginine also plays an important role in amino acid metabolism. These amino acids and proteins make up the components of the plant’s photosynthetic machinery (Ma et al., 2013; Huo et al., 2020). Therefore, under N deficiency, the application of exogenous arginine increases *Pn*, *gs*, and *Tr*, which increases the rapidity and efficiency of CO_2_ assimilation and carbohydrate production under low N stress.

The content of chl *b* was significantly affected by arginine. Previous studies have shown that chl *b* functions in the light-harvesting complex (LHC) component of plants, plays an important role in regulating the size of the photosynthetic antenna, and maintains the stability of LHCII (Yamasato et al., 2005). This indicated that arginine helps reverse the negative effect of N deficiency by maintaining the content of chl *b*. The *F_v_/F_m_* results also lead to the same conclusion. These results indicate that exogenous arginine can alleviate the degradation of chl and reduce the damage to PSII to increase photosynthetic efficiency caused by N deficiency, which enhances plant growth.

Arginine plays an important role in many biological processes. It can be broken down into intermediates that participate in the Krebs cycle (Vance et al., 2017). It also participates in the synthesis of amino acids, γ-aminobutyric acid (GABA), and NO (Majumdar et al., 2016). The content of most of the amino acids decreased under N deficiency, and N deficiency had a significant effect on amino acid synthesis. This effect was alleviated by supplementation of arginine, which increased the content of glutamate family amino acids such as the content of arginine, glutamate, proline, and ornithine. Proline is an essential amino acid that contributes to a variety of physiological and molecular responses in plants under stress (Saleem et al., 2020). Glutamate plays an important role in the synthesis of amino acids. It is a precursor in the synthesis of ornithine, as well as an intermediate in the synthesis of proline. Arginine is also a precursor in ornithine synthesis and a substrate of polyamine synthesis. The reactions of amino acids are also related to the TCA cycle, suggesting that arginine can affect the carbohydrate supply (Majumdar et al., 2015). These results were consistent with the metabolite profiling analysis. The fact that the content of amino acids changed significantly after arginine supplementation indicated that arginine is a key biochemical hub in amino acid metabolism.

Arginine affected specialized metabolites, and this might explain why the tolerance of plants to N deficiency increased. Metabolomic fingerprinting and KEGG analysis identified several significantly regulated metabolites, which were mainly divided into four groups: flavonoids, alkaloids, phenols, and amino acid derivatives. Phenols, alkaloids, and terpenoids are the main chemical substances involved in plant defense (Nguyen et al., 2020). Anthocyanins are flavonoids, which are water-soluble plant pigments with strong antioxidant activity (Meng et al., 2019). Gil et al. (2000) found that the remaining phenolic compounds only accounted for 28% of the total antioxidant activity after removing the anthocyanin fraction from pomegranate juice, indicating that anthocyanins can contribute important antioxidant effects. Phenols have been shown to be antioxidants that can remove excess reactive oxygen in cells; they also provide various benefits to human health (Shay et al., 2015; Zhang et al., 2019). In this study, the content of delphinidin-3-O-arabinoside, malvidin-3-O-arabinoside, and kaempferol-3-O-rhamnosyl (1→2) glucoside was up-regulated under LNA but down-regulated under LN. The alkaloids diethanolamine and caffeine were significantly up-regulated. The latter is known to play a role in the tolerance of viruses, bacteria, and pest insects; it is thus often used as an anti-herbivory and allelopathic agent (Kim et al., 2006, 2010). Phenols including 4-O-methylgallate were up-regulated more than 5,000 times under LNA. They are the major product of gallic acid metabolism *in vivo*. Gallic acid has antioxidant activity. A sharp increase in gallic acid was detected after arginine supplementation under N deficiency, suggesting that phenolic acid plays an important role in mediating the effects of arginine under N deficiency. The jasmonic acid content significantly increased under N deficiency and was not induced by arginine. This indicated that the absorption of exogenous arginine by *M. hupehensis* can help plants produce more antioxidant substances under N deficiency to reduce the damage caused by reactive oxygen species.

In sum, there are two possible mechanisms by which arginine alleviated the effects of N deficiency. First, arginine can promote the absorption and use of N, P, and K to compensate for the lack of N; it also can be converted into N at the same time, thereby inhibiting chl degradation to improve the efficiency of the photosynthetic system and plant growth. Second, arginine is a biochemical hub in amino acid synthesis and metabolism. Consequently, the application of exogenous arginine altered the content of a large number of amino acids in apple plants. Many intermediates participate in the urea cycle during the conversion and synthesis of these amino acids. Application of arginine affects the content of antioxidant metabolites, such as phenols, alkaloids, terpenoids, and flavonoids, which play a key role in reducing the peroxidation damage experienced by apple plants under N deficiency. The results of this study provided new insights into the mechanisms by which arginine alleviates the effects of N-deficiency stress. Additional studies are needed to further our understanding of these mechanisms.

## Data Availability Statement

The datasets presented in this study can be found in online repositories. The names of the repository/repositories and accession number(s) can be found in the article/Supplementary Material.

## Author Contributions

FM, CL, and QC conceived and designed the experiments. QC performed the experiments, analyzed the data, and wrote the manuscript. YW, ZZ, and XL prepared the materials and obtained the experimental data. All authors read and approved the manuscript.

## Conflict of Interest

The authors declare that the research was conducted in the absence of any commercial or financial relationships that could be construed as a potential conflict of interest.

## Publisher’s Note

All claims expressed in this article are solely those of the authors and do not necessarily represent those of their affiliated organizations, or those of the publisher, the editors and the reviewers. Any product that may be evaluated in this article, or claim that may be made by its manufacturer, is not guaranteed or endorsed by the publisher.

## Footnotes

1
www.r-project.org/

## Supplementary Material

Supplementary Figure 1 Concentration of total nitrogen, phosphorus, and potassium. Nitrogen in the leaf **(A)**, stem **(B)**, and root **(C)**; total potassium in the leaf **(D)**, stem **(E)**, root **(F)**, total phosphorus in the leaf **(G)**, stem **(H)**, and root **(I)** after 20 days of treatment of the four groups. Values are means of three replicates ± SD. Values not represented by the same letter are significantly different according to Tukey’s multiple-range test (*P* < 0.05).
