# Supplementary material for: Arginine Increases Tolerance to Nitrogen Deficiency in Malus hupehensis via Alterations in Photosynthetic Capacity and Amino Acids Metabolism
Source: Front Plant Sci. 2022 Jan 14;12:772086. doi: 10.3389/fpls.2021.772086 (PMC8795616; doi:10.3389/fpls.2021.772086)
Supplement: Supplementary file 2 [file Table_1.docx]

***MdTyDc* overexpression improves alkalinity tolerance in transgenic apple**

**Supplementary materials**

**TABLE. S1** Sequences of primers used in quantitative real-time RT-PCR.

| Gene | Primer sequence (5’ -3’) |
| --- | --- |
| *MDH* | F: CGTGATTGGGTACTTGGAAC  R: TGGCAAGTGACTGGGAATGA |
| *MdAMT1;2* | F: AAGCGAGGATGAGACTCAAGGG  R: GCAGGACTGGCATCATTAACAGG |
| *MdAMT2;1* | F: GTGACGATGGATCGATTGAGACTC  R: CCCGCTAACAAAATAAGAGTAATAGCT |
| *MdFd-GOGAT* | F: CGAAGGAAGAAGAAGACCACGC  R: TTGCTGGTGCCTGTTGGGTT |
| *MdNADH-GOGAT* | F: TGCCTAAGTTTATCAAGGTTATTCC  R: CTCATCTTCCTCCTCGTGCTCT |
|  |  |

**TABLE. S2** The different expression level of common metabolites between CK vs. LN and LN vs. LNA

| **Class** | **Compounds** | **Type** | | | | | |
| --- | --- | --- | --- | --- | --- | --- | --- |
|  |  | *CK vs. LN* | | | *LN vs. LNA* | | |
|  |  | *VIP* | *Fold Change* | *Type* | *VIP* | *Fold Change* | *Type* |
| Amino acids  and derivatives | L-Serine | 1.50 | 0.24 | down | 1.63 | 5.76 | up |
|  | L-Arginine | 1.49 | 0.25 | down | 1.65 | 5.29 | up |
|  | 5-Aminovaleric acid | 1.43 | 0.45 | down | 1.58 | 2.42 | up |
|  | Pipecolic acid | 1.48 | 0.25 | down | 1.60 | 3.67 | up |
|  | L-Asparagine | 1.50 | 0.03 | down | 1.65 | 49.01 | up |
|  | L-Ornithine | 1.51 | 0.03 | down | 1.65 | 52.73 | up |
|  | L-Citrulline | 1.49 | 0.24 | down | 1.64 | 3.99 | up |
|  | L-Cysteinyl-L-glycine | 1.32 | 0.28 | down | 1.45 | 4.41 | up |
|  | N-Monomethyl-L-arginine | 1.50 | 0.28 | down | 1.65 | 3.39 | up |
|  | Homoarginine | 1.49 | 0.42 | down | 1.62 | 2.39 | up |
|  | Glutathione reduced form | 1.31 | 0.31 | down | 1.22 | 2.76 | up |
|  | L-Homomethionine | 2.96 | 1.57 | up | 1.50 | 0.39 | down |
| Flavonoids | Delphinidin-3-O-arabinoside | 1.21 | 0.08 | down | 1.37 | 20.25 | up |
|  | Malvidin-3-O-arabinoside | 1.47 | 0.30 | down | 1.63 | 3.85 | up |
|  | Kaempferol-3-O-rhamnosyl (1→2) glucoside | 1.47 | 0.09 | down | 6.52 | 2.71 | up |
| Organic acids | 3-Ureidopropionic Acid | 1.34 | 0.01 | down | 1.48 | 165.01 | up |
| Phenolic acids | 4-O-Methylgallic Acid | 1.50 | 0.00 | down | 1.64 | 5057.22 | up |
| Alkaloids | Diethanolamine | 1.47 | 0.36 | down | 1.64 | 4.19 | up |
| Terpenoids | 1-Oxo-Siaresinolic acid | 1.40 | 2.75 | up | 1.59 | 0.36 | down |
|  | Myrianthic acid | 1.39 | 7.59 | up | 1.57 | 0.25 | down |
| Lipids | LysoPC 20:4 | 1.49 | 1131.60 | up | 1.10 | 0.30 | down |

**TABLE. S3** Different metabolites between CK vs. LN and LN vs. LNA

|  | Class | Compounds | VIP | Fold Change | Type |
| --- | --- | --- | --- | --- | --- |
| LN vs. LNA | Amino acids and derivatives | L-Threonine | 1.63 | 2.23 | up |
|  |  | Nicotianamine | 1.57 | 2.03 | up |
|  |  | L-Tryptophan | 1.64 | 0.42 | down |
|  | Phenolic acids | 5-O-p-Coumaroylquinic acid O-glucoside | 1.57 | 0.40 | down |
|  | Nucleotides and derivatives | β-Pseudouridine | 1.06 | 2.29 | up |
|  |  | Uridine | 1.22 | 2.01 | up |
|  | Terpenoids | Ursolaldehyde | 1.17 | 0.49 | down |
|  | Alkaloids | Caffeine | 1.24 | 4.35 | up |
|  |  | 1-Methoxy-indole-3-acetamide | 1.64 | 0.44 | down |
|  |  | Methoxyindoleacetic acid | 1.65 | 0.35 | down |
|  |  | Melatonin (N-Acetyl-5-methoxytryptamine) | 1.35 | 0.42 | down |
| CK vs. LN | Alkaloids | N-Benzylmethylene isomethylamine | 1.49 | 2.33 | up |
|  | Amino acids  and derivatives | L-Phenylalanine | 1.49 | 2.34 | up |
|  |  | L-Aspartic Acid | 1.48 | 0.46 | down |
|  |  | O-Acetylserine | 1.49 | 0.49 | down |
|  |  | N-Acetyl-L-glutamic acid | 1.46 | 0.36 | down |
|  | Flavonoids | Pelargonidin-3-O-glucoside | 1.16 | 2.28 | up |
|  |  | Cyanidin-3-O-glucoside (Kuromanin) | 1.03 | 2.60 | up |
|  |  | Hesperetin-7-O-rutinoside (Hesperidin) | 1.06 | 3.25 | up |
|  | Lignans and Coumarins | Lirioresinol A | 1.49 | 2.22 | up |
|  |  | Syringaresinol | 1.46 | 2.35 | up |
|  |  | Pinoresinol-4-O-glucoside | 1.38 | 0.48 | down |
|  | Nucleotides and derivatives | 6-Methylmercaptopurine | 1.50 | 2.34 | up |
|  |  | Cytarabine | 1.35 | 0.46 | down |
|  | Organic acids | Jasmonic acid | 1.31 | 2.20 | up |
|  |  | Isonicotinic acid | 1.47 | 0.50 | down |
|  | Others | Nicotinic acid (Vitamin B3) | 1.47 | 0.49 | down |
|  | Phenolic acids | Ferulic acid | 1.39 | 0.46 | down |
|  |  | Anthranilate-1-O-Sophoroside | 1.36 | 0.49 | down |
|  |  | Vnilloylcaffeoyltartaric acid | 1.43 | 0.49 | down |
|  | Tannins | Gallic acid | 1.50 | 0.44 | down |
|  | Terpenoids | Madasiatic acid | 1.31 | 2.59 | up |
|  |  | Madecassic acid | 1.45 | 2.85 | up |
